# Nonlinear canonical correspondence analysis and its application

**DOI:** 10.1038/s41598-023-34515-y

**Published:** 2023-05-09

**Authors:** Leru Zhou, Zhili Liu, Fei Liu, Jian Peng, Tiejun Zhou

**Affiliations:** 1 College of Resources and Environment, Xinjiang Agricultural University, Urumqi, 830052 China; 2 College of Information and Intelligence Science, Hunan Agricultural University, Changsha, 410128 China

**Keywords:** Ecology, Mathematics and computing

## Abstract

The canonical correspondence analysis (CCA) is a multivariate direct gradient analysis method performing well in many fields, however, when it comes to approximating the unimodal response of species to an environmental gradient, which still assumes that the relationship between the environment and the weighted species score is linear. In this work, we propose a nonlinear canonical correspondence analysis method (NCCA), which first determines the most appropriate nonlinear explanatory factor through two screenings by correlation and LASSO regression, and successively uses the linear regression method and the improved heuristic optimal quadratic approximation method to fit the chi-square transformation values of the response variables. Thus, our method effectively reflects the nonlinear relationship between the species and the environment factors, and a biplot is employed to visualize the effects of the later on the distribution of species. The results from applying this method over a real dataset show that the NCCA method not only maintains the advantages of the polynomial canonical correspondence analysis (PCCA) proposed by Makarenkov (2002), but also outperforms Makarenkov’s method in explaining the variance of response variables.

## Introduction

The ordination in community ecology means that the sample sites of the communities surveyed in a certain area are arranged by the similarity. The ordination axis can reflect a certain ecological gradient, which is able to explain plant species and environmental factors. In 1986, Ter Braak proposed the linear canonical correspondence analysis (LCCA) method^1^, which builds on the reciprocal averaging^2,3^. The LCCA is a direct gradient analysis method defining the coordinate axis as a linear combination of environmental factors, so that the community change may be directly related to an environmental change^1^ .

The LCCA works well under the condition of the skewed distribution of species and the high noise level^4,5^, which has become a favorite tool for ecologists^6–11^ due to its ability to correlate a data table (*Y*) of the response variables (usually are species abundance) with a second data table (*X*) of the explanatory variables (usually are environmental factors). Furthermore, the functions of the CCA have gradually been expanded from ordination to classifying^12–14^. And with the development of researches, the applications of the CCA have been expanded from the field of ecology to non-ecological fields^15–21^. For instance, in the aspect of the cell differentiation process, Masahiro Ono and Tanaka et al. studied the data sets of two transcriptomes to reveal the molecular characteristics of undefined cells by using the CCA , and finally, they visualized the T cell differentiation process^15^. Kovács Levente, Fruzsina Kézér Luca, et al. investigated the associations between the heart rate variability parameters and some housing- and individual-related variables by using the CCA method in lactating Holstein-Friesian dairy cows^16^. Cheng and Xu used the CCA to analyze the relationship between the hotel’s carbon emission effectiveness and the key indicators^17^. Santana and Alonso et al. constructed the relationship between the teaching methods and the coping of academic pressure by using the CCA^18^.

Above descriptions show that the CCA method is of good application prospect and research value. However, there are still some shortcomings with the method, one of which is its linear approximating the response of species to environmental gradients. Although the chi-square transformation of the species abundance is adopted by the algorithm, which still assumes that the relationship between the transformed response data and the environmental variables is linear^1^. As Vladimir Makarenkov et al. pointed out^22^, when modeling ecological processes, there is no sufficient reason to believe that there is a linear relationship between the changes in species assemblages and the environmental changes. For example, Austin et al. proved that there is an effective “log linear” curve relationship among the probability of the presence of eucalypt species and the altitude, the rainfall, the geology and the slope direction by using the generalized linear modeling (GLM)^23,24^. For this, many multivariate statistical analysis methods, such as canonical correlation analysis^25,26^, redundancy analysis^27^, independent component analysis^28,29^ and canonical dependency analysis^30^, have their corresponding nonlinear versions, and the linear assumption in the CCA is thus impractical. When using LCCA to analyze a problem, if the LCCA model is not very significant, but there are no other methods to use, only the LCCA method can be reluctantly used, then the results obtained must be unreliable.

In order to improve above assumption, Vladimir Makarenkov and Pierre Legendre proposed a nonlinear CCA based on the polynomial regression, they used the polynomial regression (not the multiple linear regression) to describe the relationship between the chi-square transformed response data and the explanatory variables^22^. However, the polynomial canonical correspondence analysis (PCCA) method limits the highest order of the explanatory variables in polynomials to 2, and does not include other nonlinear forms of the explanatory variables. Like the log linear relationship between the eucalyptus and the environmental variables^23,24^, this nonlinear case is unable to be effectively analyzed by the PCCA. Due to its widespread use, extending the CCA algorithm to general nonlinear relations is thus of great significance.

There are several common methods to find the nonlinear relationship between two groups of variables. The first is to look for an optimal scaling of the data by using nonlinear functions. For example, the authors divided the variables into discrete nominal, ordinal and numerical measurement levels, and adopted corresponding non-linear transformations permitted for different measurement level of each variable^25,27^. Another method is to use kernel trick, which uses a kernel function to replace the inner product operation of transformed vectors. The kernel method has been used in canonical correlation analysis and canonical independent component analysis to form their nonlinear ways^28,31^. The two methods are basically the same in principle, and both use nonlinear functions to map the observations to a new feature space. When extending CCA algorithm, we can use this non-linear idea for reference. However, the CCA is different from canonical correlation analysis or canonical independence analysis. The latter two analyses solve the problem of how to combine two groups of variables to make them most relevant or independent. One of the purposes of CCA is how to make environmental variables more fully explain species variables. This explanation is mainly tested by the significance of the regression equations. In addition, CCA needs to calculate multiple scores. After non-linear, how to calculate the score of environmental factors to reflect its non-linear impact is also a problem to be solved.

In this work, we propose a new nonlinear canonical correspondence analysis (NCCA) to solve the problem of the nonlinear relationship between the chi-square transformed response data and the environmental factors. Furthermore, we provide a method to measure the nonlinear impact of environmental factors based on the multiple correlation coefficients, and give a significance test method of the NCCA. Finally, the hunting spider dataset is selected to further illustrate the advantages of the NCCA method compared to the traditional CCA methods.

## The nonlinear canonical correspondence analysis method

Here, the *Y* is assumed to be a species matrix of size $n\times p$, where *n* is the number of sites, *p* is the number of species, and the column $y_j$ of the matrix *Y* represents the *j*-th species vector, expressed as $y_j=(y_{1j},y_{2j},\ldots ,y_{nj})^{T}$, where $y_{ij}$ represents the species abundance, presence/absence or other frequency data of species *j* observed in site *i*. And the sum of the row *i* in *Y* is denoted by $y_{i+}=\sum _{j=1}^p y_{ij}$, the sum of the column *j* in *Y* is denoted by $y_{+j}=\sum _{i=1}^n y_{ij}$, and the total sum of *Y* is denoted by $y_{++}=\sum _{i=1}^n\sum _{j=1}^p y_{ij}$. Then we define the row weight $p_{i+}=\frac{y_{i+}}{y_{++}}$, the column weight $p_{+j}=\frac{y_{+j}}{y_{++}}$ and the relative frequency $p_{ij}=\frac{y_{ij}}{y_{++}}$. With the row weights as the diagonal elements, such as $D(p_{i+})={{\,\textrm{diag}\,}}\{p_{1+}, p_{2+}, \ldots , p_{n+}\}$, a diagonal matrix is constructed. The *X* is an explanatory variables matrix of size $n\times m$, where *m* is the number of the explanatory variables, the rows represent the same sites as *Y*, and the columns of the matrix *X* represent the explanatory variables observed by the sample sites. The basic steps of the LCCA were described at length in several literature^22,32,33^. The LCCA is calculated based on a matrix $Y^*$ obtained by the following chi-square transformation

1 $$\begin{aligned} Y^*=[{\bar{y}}_{ij} ]=\left[ \frac{p_{ij}-p_{i+} p_{+j}}{\sqrt{p_{i+} p_{+j} }}\right] \end{aligned}$$

and a weighted matrix $X_t$ of the explanatory variable *X*

2 $$\begin{aligned} X_t=D(\sqrt{p_{i+}})X. \end{aligned}$$

The second step for calculating the LCCA is to calculate the regression fitting value matrix ${\hat{Y}}=X_t B$ of $Y^*$ with the multiple linear regression method

3 $$\begin{aligned} {\hat{Y}} = X_t B = X_t [(X_t)^T X_t ]^{-1} (X_t)^T Y^*, \end{aligned}$$

which reflects a linear relationship between $Y^*$ and the weighted explanatory variable $X_t$. If the LCCA model is not significant, we need to further consider whether there is a nonlinear relationship between $Y^*$ and $X_t$. Even if the LCCA model is significant, we can consider whether there is a more significant nonlinear relationship between $Y^*$ and $X_t$ than the linear relationship. Our aim is to generalize this relation to a nonlinear one. The algorithm in this section is thus designed to represent the response variable $Y^*$ as some nonlinear functions of the most relevant weighted explanatory variables. In our study, the cross term of nonlinear factors is still introduced. But for avoiding the over-fitted response variables, the number of nonlinear factors in the regression should be minimized, the reason for which is if the response variable in the regression is provided by more than $(n-1)$ explanatory variables, the over-fitting will occur, where *n* is the number of observations.

The NCCA method mainly includes the following steps.

1. Data preparation

Similar to the LCCA method, the matrix $Y^*$ is obtained from the matrix *Y* by the chi-square transformation (1). One of the differences from the LCCA is to find the nonlinear forms of environmental data significantly relating to the species distribution, and which are determined by the following process.

1. Preliminarily calculating the nonlinear factor values and the centralization.
  For some nonlinear functions $f_1$, $f_2$, $\ldots$, $f_K$, we may obtain an explanatory data matrix
  $$\begin{aligned} X_e=\left[ f_0(X),f_1(X),f_2(X),\ldots ,f_K(X)\right] , \end{aligned}$$
  where $f_0(X)=X$. By taking the column sum of $D(p_{i+})X_e$ as the weighted average ${\bar{X}}_e$, the matrix $X_e$ is centered to
  4 $$\begin{aligned} X_c=X_e-{\bar{X}}_e, \end{aligned}$$
  and then is weighted with $D(\sqrt{p_{i+}})$
  $$\begin{aligned} X_c=D(\sqrt{p_{i+}})X_c. \end{aligned}$$
2. Selecting the nonlinear environmental factors by the correlation coefficients and the significance level
Given a significance level of the correlation coefficient $\alpha$, we first calculate the correlation coefficients ${{\,\textrm{cor}\,}}(i,f_k(x_j))$ and the significance *p* values ${{\,\textrm{p}\,}}(i,f_k(x_j))$ of any column $f_k(x_j)$ in the environmental data matrix $X_c$ and any column *i* in the data matrix $Y^*$ of species, where $i = 1, 2, \ldots , p$, $j = 1, 2, \ldots , m$ and $k = 0, 1, 2, \ldots , K$. Then we select the nonlinear factors $f_k(x_j)$ with the significant correlation (${{\,\textrm{p}\,}}(i, f_k(x_j)) < \alpha$) and the absolute value of the correlation coefficient ${{\,\textrm{cor}\,}}(i,f_k(x_j))$ is greater when compared to that of the ${{\,\textrm{cor}\,}}(i,f_0(x_j))$ between the original variable $x_j$ and the species *i*.
  As we know, the over-fitting will occur if the number of the selected environmental factors is greater than $n-1$. Therefore, further selecting the nonlinear column variables by employing the absolute values of the maximum species correlation coefficients of the top $n-1-m$ from large to small is necessary.
  Under no nonlinear environmental factor being selected, we need to change a new set of nonlinear functions $f_1$, $f_2$, $\ldots$, $f_K$ or modify the significance level $\alpha$.
  If there is still no an appropriate nonlinear environmental factor selected, but several groups of different nonlinear functions are replaced repeatedly and the significance level is vary large (e.g. above 0.2), our data are not suitable for analyzing with the NCCA.
  By above selection process, a matrix $X_{expand}$ is obtained
  $$\begin{aligned} X_{expand}= [X, \text {non-linear functions of } X \text { strongly related to } Y^*]. \end{aligned}$$
  In the following processing, the matrix $X_{expand}$ is similarly centered to the expression (4).
3. Estimating the distribution of species by using the nonlinear environmental factors and the LASSO regression
  Based on the requirements of penalized regression equation, the LASSO regression^34^ is performed for each species $y_{j}^*$ with respect to $X_{w}=D(\sqrt{p_{i+}}) X_{expand}$. The factors in $X_{expand}$, which are significantly relating to the species $y_{j}^*$, are selected to form a new explanatory variable matrix $X_{expand}^{y_{j}^*}$ of the species $y_{j}^*$. At the same time, a regression coefficients vector $b^{y_{j}^*}$ with respect to the matrix $X_{w}^{y_{j}^*}=D(\sqrt{p_{i+}})X_{expand}^{y_{j}^*}$ is obtained. Therefore, the estimated species abundance ${\hat{y}}_{j}^*$ can be calculated as follows,
  5 $$\begin{aligned} {\hat{y}}_{j}^* = X_{w}^{y_{j}^*} b^{y_{j}^*} = X_{w}^{y_{j}^*} [(X_{w}^{y_{j}^*})^T X_{w}^{y_{j}^*} ]^{-1} (X_{w}^{y_{j}^*})^T y_{j}^*. \end{aligned}$$
  By deleting the same columns and retaining the different columns in different matrices $X_{expand}^{y_j^*}$ , we can obtain the new matrix $X^*$
  6 $$\begin{aligned} X^*=\cup _{y_j^*\in Y^*} X_{expand}^{y_j^*}. \end{aligned}$$
  In this variable screening process, we need to save the relationship between each column in $X^*$ and the original explanatory variable, so as to facilitate subsequent variable analysis.
  If the number *k* of elements in set $X_{expand}^{y_j^*}$ is equal to $n-1$, we skip the following step 3).
4. Considering the interaction among the explanatory variables
  It is noted that the species abundance may be affected by the interaction among the explanatory variables, which is also needed to be considered. Obviously, the estimated response variable value ${\hat{y}}_j^*$ as (5) does not reflect the interaction among the explanatory variables. But the difference $y_{j,res}^*$ between the observed and the estimated response variable value
  7 $$\begin{aligned} y_{j,res}^*=y_{j}^*-{\hat{y}}_j^* \end{aligned}$$
  reflects this interaction if it exists. By constructing the cross term matrix and using the stepwise regression method, we can obtain the cross factor estimation of the residual $y_{j,res}^*$. However, the large number of cross terms make it difficult to control the number of cross terms in the regression estimation expression, this method is thus not widely used in practical calculation. V.Makarenkov provided a heuristic optimal quadratic approximation method aimming at above residuals^22^. We modify the method by our requirements as follows. We consider only the cross term and not the square term in constructing the design matrix.
  For this reason, for each pair of columns *r* and *s* in $X_{expand}^{y_j^*}$, by calculating a simple linear regression of the residual vector $y_{j,res}^*$ on cross term $x_r x_s$, where $x_r$, $x_s$ are two columns in $X_{expand}^{y_j^*}$, we obtain the regression estimation ${\hat{y}}_{j,res}^{*rs}$ of the residual vector $y_{j,res}^{*}$ on the variables $x_r$ and $x_s$ as follows
  $$\begin{aligned} {\hat{y}}_{j, res}^{*rs} = c_1^{rs} x_r x_s+c_0^{rs}. \end{aligned}$$
  Then, we calculate the adjusted determination coefficient $R_{adj}^2(r,s)$,
  8 $$\begin{aligned} R_{adj}^2(r,s)=1-\frac{n-1}{n-k-1}\frac{\sum _{i=1}^n (y_{j,res,i}^{*}-{\hat{y}}_{j,res,i}^{*rs})^2}{\sum _{i=1}^n(y_{j,res,i}^{*}-{\bar{y}}_{j,res}^*)^2}, \end{aligned}$$
  where *k* represents the number of elements of set $X_{expand}^{y_j^*}$, ${\bar{y}}_{j,res}^*$ is the mean of vectors ${y}_{j,res}^*$, and $y_{j,res,i}^{*}$ (${\hat{y}}_{j,res,i}^{*rs}$) represents the *i*-th element of the vector $y_{j,res}^{*}$ (${\hat{y}}_{j,res}^{*rs}$).
  Repeating this process for each column pair (*r*, *s*) of the matrix $X_{expand}^{y_j^*}$, and comparing all the new determination coefficients with the original one, then we can obtain the columns pair (*r*, *s*) with the largest and better determination coefficient $R_{adj}^2(r,s)$, that is, after introducing their cross term, the adjusted determination coefficient is the largest and higher than that when they are not introduced. By combining the two selected explanatory variables $x_r$ and $x_s$, a new join explanatory variable vector $x_{join}$ is formed as follows,
  9 $$\begin{aligned} x_{join}=b^{y_{j}^*}_rx_r+b^{y_{j}^*}_sx_s+c_1^{rs}x_rx_s+c_0^{rs}. \end{aligned}$$
  The new combined explanatory variable $x_{join}$ includes the linear and cross term contributions of the variables $x_r$ and $x_s$ fitting $y_j^*$. By deleting the columns *r* and *s* and adding a new column vector $x_{join}$, the number of columns of the matrix $X_{expand}^{y_j^*}$ is one less than before.
  We repeat the previous process in step (2)–(3) until the matrix $X_{expand}^{y_j^*}$ is converted to a matrix with only one column or no columns pair meeting the above conditions is found.
  Finally, by calculating a linear regression of $y_{j}^*$ on the final matrix $X_{expand}^{y_j^*}$, we obtain the estimated value ${\hat{y}}_j^*$ of the species $y_{j}^*$.
  For each species variable $y_j^*$ ($j=1, 2, \ldots , p)$, repeating above steps (2) and (3) may get the regression estimations of these variables. Finally, the regression estimation matrix ${\hat{Y}}$ is formed by
  10 $$\begin{aligned} {\hat{Y}}=({\hat{y}}_1^*, {\hat{y}}_2^*, \cdots , {\hat{y}}_p^*). \end{aligned}$$
  Obviously, the final regression fitting value ${\hat{y}}_j^*$ is a linear combination of some original environmental factors, some nonlinear functions of the original environmental factors, and some of their cross terms. Therefore, ${\hat{Y}}$ reflects the nonlinear effect of the environmental factors on the species.
  The following steps are basically the same as those of the LCCA, including the calculation of the eigenvalues and the eigenvectors of the covariance matrix $S_{{\hat{Y}}^T {\hat{Y}}}={\hat{Y}}^T {\hat{Y}}$, the calculations of the site scores, the species scores and the ordination axis scores of different scale types, which can be referred to the equations (C.6)–(C.11) of APPENDIX C in the literature^22^. But in order to reflect the nonlinear influence of the explanatory variables, the calculation methods of the explanatory variable scores are different from the LCCA.

## Representing the nonlinear terms of the explanatory variables with biplots

Each column in the matrix $X^*$ of the expression (6) represents a selected explanatory variable or a nonlinear term. When drawing a biplot, we need to reflect the role of all the column variables of $X^*$.

There are the following two approaches for calculating the explanatory variable scores.

Strategy (1) If the matrix $X^*$ does not contain too many variables (the columns of $X^*$), the weighted correlation coefficients $R_{Z,x}$ of any variable *x* in the matrix $X^*$ and the ordination score *Z* of each ordination axis can be calculated by the formula (C.12) of APPENDIX C in the literature^22^. Using these weighted correlation coefficients as the coordinates of the arrows in the biplot, the explanatory variable *x* or the nonlinear factor can be drawn. In this way, if there are too many variables in $X^*$, then too many arrows may present in the biplot, which can lead to the crowding. Under this case, the following second approach can be considered.

Strategy (2) The second approach is only drawing the arrows of the original explanatory variables, the position of an arrow is determined by *x*, and its nonlinear components. Let *x* be an original explanatory variable in *X* and $f_1 (x)$, $f_2 (x)$, $\ldots$, $f_a (x)$ are its nonlinear components respectively corresponding to the $i_1$, $i_2$, $\ldots i_a$ columns of $X^*$, that is, $x_{i_j}=f_j (x_i)$ is the $i_j$ column of $X^*$, we denote ${\bar{x}} = \{x, f_1 (x), f_2 (x), \ldots , f_a (x)\}$. The multiple correlation coefficient of the explanatory variable *x* and the ordination score *Z* of each ordination axis is calculated as follows,

11 $$\begin{aligned} R_{(Z,{\bar{x}})}={{\,\textrm{sign}\,}}(r_{(Z,x)})\sqrt{R_{(Z,{\bar{x}})}^T R_{({\bar{x}},{\bar{x}})}^{-1} R_{(Z,{\bar{x}})}}, \end{aligned}$$

where $r_{(u,v)}$ is the correlation coefficient of two vectors *u* and *v*, the matrix $R_{(Z,{\bar{x}})} =[r_{(Z,x)},r_{(Z,f_1 (x))},\cdots , r_{(Z,f_a (x))} ]^T$, $R_{({\bar{x}}, {\bar{x}})}$ is of the following form,

12 $$\begin{aligned} R_{({\bar{x}},{\bar{x}})}=\left[ \begin{array}{cccc} 1 &{} r_{(x,f_1 (x) )} &{} \cdots &{} r_{(x,f_a (x) )}\\ r_{(f_1 (x),x)} &{} 1 &{} \cdots &{} r_{(f_1 (x),f_a (x) )}\\ \cdots &{} \cdots &{} \cdots &{} \cdots \\ r_{(f_a (x),x)} &{} r_{(f_a (x),f_1 (x) )} &{} \cdots &{} 1 \end{array} \right] . \end{aligned}$$

The multiple correlation coefficient between the explanatory variable *x* and each coordinate axis is used as the coordinate of the arrow of the explanatory variable *x* in the biplot. Thus the arrow in the biplot reflects the nonlinear components of the explanatory variable *x*.

Similar to the LCCA, there are two scaling methods for drawing a biplot: scaling type 1 and scaling type 2 (^22^, see APPENDIX C). For scaling type 1, the coordinate of explanatory variables in the biplot still needs to multiply above multiple correlation coefficient by a weight

13 $$\begin{aligned} c_k=\sqrt{\frac{\lambda _k}{\sum \lambda _k }}, \end{aligned}$$

where $\lambda _k$ is the eigenvalue of the covariance matrix $S_{{\hat{Y}}^T}$, and the subscript *k* represents the *k*-th ordination axis.

## Significance test in the NCCA

The permutation test is employed to examine the validity of our method. In the permutation test, a statistic is recalculated on every sampling by randomly changing the position of the tested element, so that the simulated statistical values constitute a reference distribution. Now, the position of the statistical value of the current actual data in the reference distribution can be calculated, and the *p* value of the actual statistical value is the probability statistical value of the position of the actual statistical value^35^.

The null hypothesis $H_0$ for testing the significance of the NCCA is to assume that there is no nonlinear relationship between the response variables and the explanatory variables, that is, there is no nonlinear relationship between the variables in the matrix $X_{expand}$ and $Y^*$. Similar to the test process discussed by Vladimir Makarenkov and Pierre Legendre^22^, the pseudo *F* statistic is defined,

14 $$\begin{aligned} F=\frac{{{\,\textrm{var}\,}}({\hat{Y}})}{{{\,\textrm{var}\,}}(Y^*)-{{\,\textrm{var}\,}}({\hat{Y}})}, \end{aligned}$$

where ${{\,\textrm{var}\,}}(U)$ denotes the variance of *U*. The permutation test process of the NCCA is as follows.

1. For a real data set, using the NCCA method to obtain ${\hat{Y}}$, and calculating with the expression (14) the pseudo *F* statistic.
2. Permuting the rows of matrix $Y^*$, and getting a matrix $Y_{perm}^*$.
3. For the data set $Y_{perm}^*$ and $X_{expand}$ (unpermuted), using the NCCA method to obtain ${\hat{Y}}_{perm}$.
4. Calculating with the expression (14) the pseudo $F_{perm}$ statistic for the permuted data $Y_{perm}^*$ and ${\hat{Y}}_{perm}$.
5. Calculating the occurrence frequency *p* of event $\{F_{perm} > F\}$ and the standard deviation $\delta _p$ of *p* in the previous $N_1$ samplings.
6. If the standard deviation $\delta _p$ is less than a given very small threshold $\epsilon$, stopping permutation sampling, otherwise repeating steps (2)–(5) *N* times (for example, $N=999$).

Finally making the test conclusion. If *p* is less than a given significance level $\alpha$ (e.g., 0.01 or 0.05), rejecting the null hypothesis $H_0$^36^, and considering the NCCA model is significant. Otherwise, accepting the null hypothesis $H_0$.

If both the NCCA and other CCA models are significant, then it is necessary to determine which model is more suitable to describe and analyze the current data. Also, the permutation test can be used to evaluate the variance difference between the NCCA model and other CCA models. Now, the null hypothesis $H_0$ is that there is no difference between the two CCA methods. And the pseudo *F* statistic for this difference is

15 $$\begin{aligned} F=\frac{{{\,\textrm{var}\,}}({\hat{Y}}_{NCCA})-{{\,\textrm{var}\,}}({\hat{Y}}_{other\, CCA})}{{{\,\textrm{var}\,}}(Y^* )-{{\,\textrm{var}\,}}({\hat{Y}}_{NCCA})}. \end{aligned}$$

The test process is consistent with the previous steps (1)–(6). The slight difference is that after each permutation, the species estimations of the two CCA models, ${\hat{Y}}_{NCCA}$ and ${\hat{Y}}_{other\,CCA}$, need to be calculated.

## Numerical simulation

The purpose of this section is to analyze the errors of permutation test results described in the previous section and discuss whether it can provide an effective significance testing. When there is no relationship between *X* and $Y^*$, if the null hypothesis is rejected by the permutation test, the type I error occurs, or if the null hypothesis is accepted when there is a nonlinear relationship between *X* and $Y^*$, the type II error occurs. We will still analyze whether this method has a good ability to distinguish between linear and nonlinear relationships. For this reason, we will use a large number of computer simulation data to obtain the occurrence frequencies of two types of errors of NCCA in the permutation test, so as to illustrate that the NCCA method is correctly-sized and powerful. If the occurrence rates of two types of errors are low, it indicates that the NCCA method is correctly sized and has a high power. We use the first canonical correlation coefficient to measure the extent of correlation between the generated two sets of data *X* and $Y^*$.

We firstly show the NCCA model has correct type I error. When data *X* and $Y^*$ are generated randomly and have a low first canonical correlation coefficient, there is no relationship between them with high probability. Therefore, the null hypothesis should be admitted. If it is denied, the type I error has occurred. In order to evaluate the occurrence frequency of the type I error in the permutation test, the following numerical simulation process is designed.

Let $Y=(Y_1,Y_2,\ldots ,Y_p)$ be a $n \times p$ matrix of the response variables and $X=(X_1.X_2,\ldots ,X_m)$ be $n \times m$ matrix of the explanatory variables.

Step (1) Randomly generating two matrices *Y* and *X*, where $Y_i$ and $X_j$ obey the standard normal distribution ($X_j \sim N(0,1)$,$Y_i \sim N(0,1)$), respectively, and shifting them to a non-negative range. Calculating the first canonical correlation coefficient of *X*and $Y^*$. If it is greater than a threshold value of the correlation coefficient, two new matrices *X* and *Y* will be generated again.

Step (2) Analyzing data sets *Y* and *X* using NCCA, respectively.

Step (3) Testing the significance of the relationship between *Y* and *X* with the permutation test at different significance levels $\alpha$, and accumulating the number of times of the event in which NCCA rejects the null hypothesis, respectively, $k_l$, $k_p$, $k_n$.

Step (4) Repeating steps (1) to (3) *N* times.

Step (5) Calculating the rejection rate of the null hypothesis or the frequency of the type I error, respectively, $k_l/N$, $k_p/N$, $k_n/N$.

Our numerical simulation takes the paramters $n=30$, $p=4$, $m=2$, $N=1000$, and performs 499 permutation tests. Following by above steps, we obtain the occurrence frequencies of type I error through $N=1000$ experiments at different significance levels $\alpha =0.01, 0.02, 0.03, \ldots , 0.20$ and five thresholds of the first canonical correlation coefficients $r=0.15, 0.25, 0.361, 0.45, 0.55$ and 0.6, which are shown in Table 1.

**Table 1:** The occurrence frequencies of type I error of the NCCA model.

Significance level	The occurrence frequencies of type I error based on the threshold of the first canonical correlation coefficient *r*					
	$r=0.15$	$r=0.25$	$r=0.361$	$r=0.45$	$r=0.55$	$r=0.6$
0.01	0.000	0.000	0.000	0.001	0.004	0.013
0.02	0.001	0.001	0.002	0.003	0.010	0.023
0.03	0.001	0.001	0.003	0.006	0.016	0.031
0.04	0.002	0.002	0.005	0.012	0.022	0.041
0.05	0.002	0.002	0.005	0.013	0.032	0.044
0.06	0.002	0.003	0.005	0.018	0.036	0.057
0.07	0.002	0.003	0.008	0.022	0.039	0.065
0.08	0.002	0.003	0.008	0.029	0.048	0.075
0.09	0.003	0.003	0.009	0.033	0.059	0.080
0.10	0.004	0.005	0.011	0.038	0.063	0.086
0.11	0.005	0.005	0.012	0.039	0.068	0.094
0.12	0.005	0.005	0.014	0.044	0.072	0.103
0.13	0.005	0.007	0.016	0.049	0.082	0.116
0.14	0.005	0.007	0.018	0.053	0.088	0.130
0.15	0.005	0.009	0.025	0.056	0.096	0.134
0.16	0.008	0.011	0.026	0.061	0.103	0.145
0.17	0.009	0.011	0.029	0.067	0.109	0.158
0.18	0.009	0.011	0.032	0.071	0.114	0.164
0.19	0.011	0.013	0.034	0.077	0.122	0.167
0.20	0.011	0.013	0.035	0.085	0.130	0.176

Table 1 shows that the frequencies of type I error for the NCCA model are lower than the corresponding significance levels when the threshold of the first canonical correlation coefficients $r\le 0.55$. Note that the critical value of the correlation coefficient with a degree of freedom of 28 at the significance level of 0.05 is 0.361. That is say, for two sets of randomly generated data, even if their first canonical correlation coefficient exceeds the critical value by some, the NCCA method can still be correctly sized. Therefore, the simulation shows that NCCA generally does not build a nonlinear model for two sets of unrelated data.

Next, we will discuss the type II error of NCCA. When there is a nonlinear relationship between species abundances and environmental factors, whether the NCCA can correctly find this relationship is a problem reflecting its power. The numerical simulation method is also used in this problem. By generating a large number of data *Y* and *X* with some nonlinear relationship, we verify the ability of NCCA to correctly identify this relationship by permutation test. At this case, the rejecting the null hypothesis during the test indicates that NCCA has made a correct judgment. After accumulating the number of rejecting null hypothesis in all experiments, we get the rejecting frequency, that is, the frequency of NCCA correctly identifying the nonlinear relationship. The higher the frequency of rejecting null hypothesis, the stronger the power of NCCA. The basic simulation steps are similar to the above calculation of the frequency of type I error, but the difference is the first step. When generating matrix *Y*, it should be determined according to some nonlinear functions and noise, that is, $Y=f_1(X)+f_2(X)+\cdots +f_k(X)+c\sigma$, where $f_i(X)$ is a nonlinear function of *X*, $\sigma$ is the noise, which obeys a *p*-dimensional standard normal distribution, *c* is the standard deviation of noise which affects the extent of correlation between *X* and *Y*.

In order to facilitate the analysis of the next section, we take the regression equations in Table 6 as the functions that generate $Y_i$, and the relevant parameters are also the same as in the next section, namely $n=28$, $p=12$, $m=4$. The standard deviation of noise $c=0.02$. To illustrate the advantages of the NCCA method, we also use LCCA and PCCA to analyze the data generated each time. The simulation results show that when the significance level is 0.01, the frequency of rejecting null hypothesis for the nonlinear relation using NCCA method reaches $95\%$ in $N=1000$ random experiments. When the significance level increases from 0.01 to 0.2, the corresponding frequency of rejection also further increases (see the first column on the right of Table 2). That is to say, the NCCA method has a high probability of believing that there is a certain relationship between data *Y* and *X*. Therefore, the NCCA method has a strong power to discover nonlinear relationship. However, for the data generated by the nonlinear functions, the rejection frequencies of LCCA and PCCA are also so high (see the second and third columns on the right of Table 2), which indicate that LCCA or PCCA mistakenly identifies the nonlinear relationship as a linear or polynomial relationship in most cases. Therefore, the relationship between the environment and species or between the environment and the sample site revealed by LCCA/PCCA is unreliable. If there is no NCCA method, the nonlinear relationship will be incorrectly analyzed by LCCA or PCCA. In addition, we calculated the frequencies of rejecting null hypothesis under different noise standard deviations $c=0.004$, $c=0.01$, $c=1$, $c=10$,$c=30$ and $c=65$ (see Table 3). The results show that the rejection frequencies increase with the decrease of the noise standard deviation *c*.

**Table 2:** The frequency of rejecting null hypothesis for the three kinds of CCA methods.

Significance level	Linear relation			Polynomial relation			Nonlinear relation		
	LCCA	PCCA	NCCA	LCCA	PCCA	NCCA	LCCA	PCCA	NCCA
0.01	0.870	0.805	0.850	0.918	0.900	0.925	0.968	0.766	0.950
0.02	0.893	0.841	0.871	0.937	0.925	0.949	0.981	0.820	0.965
0.03	0.908	0.854	0.890	0.945	0.939	0.963	0.988	0.852	0.971
0.04	0.913	0.873	0.905	0.954	0.945	0.968	0.993	0.882	0.98
0.05	0.920	0.884	0.907	0.958	0.950	0.970	0.993	0.898	0.984
0.06	0.921	0.889	0.913	0.959	0.953	0.972	0.994	0.910	0.986
0.07	0.925	0.895	0.920	0.960	0.958	0.975	0.996	0.920	0.986
0.08	0.927	0.898	0.925	0.961	0.962	0.977	0.999	0.931	0.986
0.09	0.929	0.908	0.931	0.963	0.967	0.980	0.999	0.939	0.987
0.10	0.933	0.912	0.935	0.965	0.968	0.982	0.999	0.949	0.988
0.11	0.935	0.917	0.937	0.967	0.968	0.983	0.999	0.957	0.989
0.12	0.936	0.921	0.938	0.971	0.972	0.984	0.999	0.971	0.989
0.13	0.938	0.922	0.942	0.971	0.973	0.984	1	0.973	0.990
0.14	0.941	0.924	0.943	0.973	0.974	0.984	1	0.975	0.991
0.15	0.945	0.925	0.945	0.975	0.975	0.985	1	0.979	0.992
0.16	0.948	0.927	0.945	0.977	0.978	0.985	1	0.983	0.993
0.17	0.951	0.927	0.946	0.977	0.980	0.987	1	0.986	0.994
0.18	0.954	0.929	0.947	0.977	0.980	0.987	1	0.987	0.994
0.19	0.957	0.931	0.948	0.979	0.980	0.988	1	0.987	0.995
0.20	0.961	0.933	0.950	0.980	0.980	0.988	1	0.987	0.996

**Table 3:** The effect of noise on the performance of three CCA methods.

Significance level	The rejection frequency with noise intensity *c*							
	$c=0.01$	$c=0.05$	$c=1$	$c=10$	$c=30$	$c=55$	$c=65$	$c=100$
0.01	0.938	0.953	0.952	0.958	0.934	0.718	0.623	0.363
0.02	0.956	0.969	0.962	0.966	0.961	0.823	0.724	0.448
0.03	0.966	0.974	0.964	0.975	0.969	0.847	0.780	0.507
0.04	0.976	0.977	0.968	0.980	0.974	0.866	0.807	0.546
0.05	0.976	0.979	0.971	0.984	0.980	0.883	0.833	0.592
0.06	0.980	0.981	0.974	0.984	0.982	0.897	0.856	0.622
0.07	0.982	0.984	0.977	0.986	0.985	0.911	0.865	0.638
0.08	0.983	0.987	0.982	0.987	0.986	0.920	0.877	0.664
0.09	0.986	0.987	0.985	0.987	0.986	0.926	0.889	0.689
0.10	0.989	0.990	0.986	0.989	0.991	0.936	0.895	0.699
0.11	0.990	0.990	0.987	0.989	0.994	0.938	0.900	0.717
0.12	0.993	0.991	0.988	0.990	0.994	0.946	0.908	0.730
0.13	0.994	0.991	0.990	0.990	0.995	0.951	0.913	0.749
0.14	0.995	0.992	0.992	0.991	0.996	0.953	0.916	0.763
0.15	0.995	0.993	0.992	0.992	0.996	0.957	0.920	0.775
0.16	0.995	0.994	0.993	0.994	0.997	0.958	0.928	0.784
0.17	0.996	0.994	0.995	0.995	0.997	0.960	0.934	0.793
0.18	0.998	0.996	0.997	0.995	0.997	0.965	0.939	0.801
0.19	0.998	0.997	0.998	0.995	0.997	0.968	0.943	0.808
0.20	0.998	0.997	0.999	0.998	0.999	0.971	0.944	0.815

We also use three CCA methods to analyze the data generated by the linear function $Y=b_0+b_1X+c\sigma$ and the cubic polynomial with cross terms $Y=b_1X+b_2X^2+b_3X^3+b_4X_1X_2+c\sigma$, where $\sigma$ obeys standard normal distribution, $c=0.02$. The frequencies of rejecting null hypothesis are also listed in the second-seventh columns of Table 2. It can be seen from Table 2 that when data Y is randomly generated from linear function or polynomial, the rejection frequencies of LCCA or PCCA is relatively high, which reflect their high power to find linear or polynomial relationships. However, due to the fact that the model established by NCCA includes linear and polynomial terms, the rejection frequencies of NCCA is also high, which means it can also detect this linear or polynomial relationship. According to the difference significance test method introduced in the previous section, the frequencies of rejecting null hypothesis for the difference of NCCA with LCCA and that of NCCA with PCCA all reaches $100\%$.

The various settings considered in the numerical simulation are summarized in Table 4, where *N*(0, 1) represents the standard normal distribution, and $b_i$ obeys the uniform distribution on the interval [4, 5].

**Table 4:** The various settings considered in the numerical simulations.

Relation	Data X	Data Y	Parameters *n*, *p*, *m* and *N*
No relation	$X_j \sim N(0,1)$	$Y_i \sim N(0,1)$	$n=30$, $p=4$, $m=2$ and $N=1000$
Linearity	$X_j \sim N(0,1)$	$Y=b_0+b_1X+c\sigma$	$n=30$, $p=4$, $m=2$ and $N=1000$
Polynomial	$X_j \sim N(0,1)$	$Y=b_1X+b_2X^2+b_3X_1X_2+c\sigma$	$n=30$, $p=4$, $m=2$ and $N=1000$
Nonlinearity	$X_j \sim N(0,1)$	$Y=f(X)+c\sigma$, where *f*(*X*) are the regression functions in Table 6	$n=28$, $p=12$, $m=4$ and $N=1000$

## Experiments

Now, we demonstrate our approach through real experiments.

### The hunting spider dataset

The research on the hunting spider distribution was a part of 60-weeks extensive ecological project carried out by researchers from the department of animal ecology of Leiden University in the dune area “Meijendel” in the Hague during the period of 1969–1970. These researchers dug up 100 pitfalls with a radius of 1 m to capture spiders, and 12,156 hunting spiders from 12 species in total were captured in 60 weeks. Twenty-six environmental characteristics on the soil, the vegetation and the light around the 28 pitfall sites were measured. For the experimental data, please refer to^37^, Table I to Table IV]. In 1986, Ter Braak applied the hunting spider data set to the CCA method^1^. In 2002, Vladimir Makarenkov and Pierre Legendre also used the data set to verify the PCCA method^22^ . Subsequently, other scholars also conducted various analyses over the data set. By using the principal component analysis and the canonical correlation analysis methods, Van der Aart and Smeenk–Enserink found that the number of spider species has a very strict, well defined and reproducible relationship with a major environmental factor (a principal component)^37^. They also established the quadratic regression equations between the number of each kind of spiders and the principal components (^37^, Fig. 5 and Fig. 6). However, by the analysis of^37^, Fig. 5], the relationship between the number of spiders and the principal components is more complicated, which involves the polynomial relationships and other nonlinear relationships. Therefore, we apply the NCCA method to this hunting spider distribution data set^22,37^, then compare the results of the NCCA to that of the LCCA and the PCCA and discuss these results.

From the 26 environmental factors, the authors of the literatures^22,37^ selected four environmental variables on soil, vegetation and light: the soil water content ($x_1$), the reflection ($x_2$), the calamagrostis coverage ($x_3$) and the corynephorus coverage ($x_4$). For facilitating the comparison with the PCCA, we also select the four environmental variables.

The data set contains several nonlinear species-environment relationships. After preprocessing the original data, the correlation coefficients between the species and the corynephorus coverage or its three special nonlinear terms are shown as Table 5, from which we can see that all species except $s_2$ have stronger correlations with the nonlinear terms of the environmental factors when compared with the original environmental factor data. The correlation between the species $s_3$ and the corynephorus coverage ($x_4$) is not significant, but the correlations between the species $s_3$ and three nonlinear terms is significant (the absolute values of correlation coefficient ($-0.6596$, 0.5296, 0.4463) are all greater than the critical value 0.3739). The canonical correlation coefficients between the four original environmental variables *X* after centralization and $Y^*$ are 0.9879, 0.9736, 0.8251, 0.6760, while the first four canonical correlation coefficients of $X^*$ obtained from the non-linear transformation of *X* in the following subsection become 1, 1, 1, and 0.9995. This indicates that the relationship between $Y^*$ and nonlinear environmental factors is significant, and it is appropriate to analyze them using the NCCA method. Van der Aart and Smeenk-Enserink discussed this property^37^ . We will further discuss the nonlinear relationships in the data set.

**Table 5:** The correlation coefficients between the species and the corynephorus coverage or its three nonlinear terms.

Species	$x_4$	$(x_4+1)^{-3}$	$\log (x_4+1)$	$\sqrt{x_4+1}$
$s_1$	0.5767	− 0.6948	0.6812	0.6450
$s_2$	− 0.0493	0.2128	− 0.1670	− 0.1179
$s_3$	0.3425	− 0.6596	0.5296	0.4463
$s_4$	− 0.2123	0.2076	− 0.2138	− 0.2155
$s_5$	0.1606	− 0.5009	0.3726	0.2714
$s_6$	− 0.3181	0.3242	− 0.3282	− 0.3269
$s_7$	− 0.2502	0.2766	− 0.2716	− 0.2646
$s_8$	0.7479	− 0.5639	0.6567	0.7088
$s_9$	− 0.3197	0.3150	− 0.3233	− 0.3252
$s_{10}$	− 0.4201	0.4485	− 0.4497	− 0.4417
$s_{11}$	− 0.7363	0.8137	− 0.8052	− 0.7831
$s_{12}$	− 0.4518	0.4691	− 0.4727	− 0.4683

The critical value of the correlation coefficient with degree of freedom 26 is 0.3739 at the significance level of 0.95.

### The results of the NCCA

By taking five nonlinear functions $f_1(x)=x^2$, $f_2(x)={(x+1)}^{-3}$, $f_3(x)=\log (x+1)$, $f_4(x)=\sin (x)$ and $f_5(x)=\sqrt{x+1}$, the significance level of correlation coefficient $\alpha =0.01$, we obtained the matrix $X_{expand}$. By LASSO regression, we obtained the nonlinear regression models and the adjusted determination coefficients of regression equations listed in the second and the third columns of Table 6, and the matrix $X^*$ which includes four environmental factors $x_1$, $x_2$, $x_3$, $x_4$, and 13 nonlinear environmental factors $x_1^2$, $x_2^2$, $x_3^2$, ${(x_1+1)}^{-3}$, ${(x_2+1)}^{-3}$, ${(x_4+1)}^{-3}$, $\log {(x_1+1)}$, $\log {(x_2+1)}$, $\log {(x_4+1)}$, $\sqrt{x_1+1}$, $\sqrt{x_2+1}$, $\sqrt{x_3+1}$ and $\sqrt{x_4+1}$. After considering the interaction among the environmental factors, the estimation ${\hat{Y}}$ of $Y^*$ is obtained. Now, the adjust determination coefficients of the regression estimations have been generally improved, which are listed in the last column of Table 6, of which species $s_2$, $s_4$ and $s_7$ have the greatest improvements.

**Table 6:** The nonlinear regression modeling based on the spider species data $Y^*$ and the environmental factors.

Species	Regression models	${\textbf{R}}_{adj}^2$ of regression models	${\textbf{R}}_{adj}^2$ of final models
$s_{1}$	− 0.00075 − 0.24875$\ln \left( {x_{1}}+1\right)$ + 0.03108$\sqrt{{x_{2}}+1}$ + 0.04454$\ln \left( {x_{4}}+1\right)$ + 56.32915$\frac{1}{{\left( {x_{1}}+1\right) }^3}$	0.78669	0.80952
$s_{2}$	− 0.00228 + 0.02710${x_{1}}$ + 0.00669${x_{2}}$ − 0.00042${x_{3}}$ + 0.00077${x_{4}}$ − 0.00027$x_{1}^2$	0.19134	0.47269
$s_{3}$	0.00027 − 0.02696${x_{4}}$ + 0.00197$x_{1}^2$ + 0.00002$x_{3}^2$ − 2.09671$\ln \left( {x_{1}}+1\right)$ − 0.10250$\sqrt{{x_{2}}+1}$ − 44.07641$\frac{1}{{\left( {x_{1}}+1\right) }^3}$ − 1.02456$\frac{1}{{\left( {x_{4}}+1\right) }^3}$	0.69921	0.72230
$s_{4}$	− 0.00181 + 0.00546${x_{1}}$ − 0.00092${x_{2}}$ + 0.00028${x_{3}}$ + 0.00016${x_{4}}$	0.09586	0.52954
$s_{5}$	− 0.00026 − 2.41829${x_{1}}$ − 1.35215${x_{2}}$ + 0.01643${x_{3}}$ + 0.25302${x_{4}}$ + 0.01004$x_{1}^2$ + 0.00580$x_{2}^2$ − 0.00012$x_{3}^2$ − 22.28416$\ln \left( {x_{1}}+1\right)$ − 10.08670$\ln \left( {x_{2}}+1\right)$ + 3.80500$\ln \left( {x_{4}}+1\right)$ + 28.20421$\sqrt{{x_{1}}+1}$ + 14.55393$\sqrt{{x_{2}}+1}$ − 0.07259$\sqrt{{x_{3}}+1}$ − 4.34958$\sqrt{{x_{4}}+1}$ − 65.56818$\frac{1}{{\left( {x_{1}}+1\right) }^3}$ − 10.26494$\frac{1}{{\left( {x_{2}}+1\right) }^3}$ + 0.64984$\frac{1}{{\left( {x_{4}}+1\right) }^3}$	0.94541	0.95262
$s_{6}$	0.00148 − 0.00303${x_{2}}$ + 0.00002$x_{2}^2$ + 0.03299$\sqrt{{x_{3}}+1}$	0.23524	0.27834
$s_{7}$	0.00094 − 0.01634${x_{1}}$ + 0.00301${x_{2}}$ − 0.00009${x_{3}}$ − 0.00329${x_{4}}$ − 0.18361$\ln \left( {x_{2}}+1\right)$ + 10.29795$\frac{1}{{\left( {x_{2}}+1\right) }^3}$	0.35049	0.69434
$s_{8}$	− 0.00159 + 0.16602$\ln \left( {x_{2}}+1\right)$ + 0.51059$\ln \left( {x_{4}}+1\right)$ − 0.00013$x_{1}^2$ − 0.00003$x_{3}^2$ − 0.10582$\sqrt{{x_{4}}+1}$ − 86.00328$\frac{1}{{\left( {x_{1}}+1\right) }^3}$	0.81882	0.82967
$s_{9}$	− 0.00229 − 0.00379${x_{2}}$ + 0.00532${x_{3}}$ + 0.01916$\sqrt{{x_{1}}+1}$	0.49288	0.58257
$s_{10}$	− 0.00077 − 0.00289${x_{1}}$ − 0.00270${x_{2}}$ − 0.01164${x_{3}}$ + 0.00109${x_{4}}$ + 0.19926$\sqrt{{x_{3}}+1}$ − 0.02821$\frac{1}{{\left( {x_{4}}+1\right) }^3}$	0.70521	0.76148
$s_{11}$	0.00251 + 0.00546${x_{2}}$ + 0.01095${x_{4}}$ − 0.00082$x_{1}^2$ + 0.00003$x_{2}^2$ + 0.00008$x_{3}^2$+ 0.98200$\ln \left( {x_{1}}+1\right)$ − 0.21694$\ln \left( {x_{4}}+1\right)$ − 0.15699$\sqrt{{x_{3}}+1}$ + 42.54762$\frac{1}{{\left( {x_{1}}+1\right) }^3}$ − 1.81067$\frac{1}{{\left( {x_{2}}+1\right) }^3}$ + 0.53053$\frac{1}{{\left( {x_{4}}+1\right) }^3}$	0.76466	0.80463
$s_{12}$	− 0.00209 + 0.00019$x_{1}^2$ − 0.10112$\ln \left( {x_{2}}+1\right)$ − 0.02839$\ln \left( {x_{4}}+1\right)$ − 0.03960$\frac{1}{{\left( {x_{4}}+1\right) }^3}$	0.43083	0.53130

The standard error between the estimated value of $Y^*$ based on these regression equations in Table 6 and $Y^*$ is 0.0058. According to the discussion in the Section of “Numerical simulation”, the NCCA method has excellent performance under this noise intensity. The results of the NCCA based on the scaling type 2 are shown in Supplementary Table S1 online. A total of 12 canonical axes are obtained by using the NCCA method. Supplementary Table S1 shows the first six canonical axes, where the eigenvalue is the variance of each canonical axis, and the variance ratio is the ratio of the eigenvalue to the total variance of $Y^*$ (The total variance is equal to 1.92296), the cumulative variance ratio represents the cumulative value of the variance ratio. The first four canonical axes account for $75.38650\%$ of the total variance of $Y^*$, which is significantly larger than $43.80558\%$ of the four canonical axes based on the LCCA, and the first two canonical axes also explain $54.80844\%$ of the total variance. Therefore, an effective biplot ordination can be obtained in two-dimensional space.

**Figure 1:**
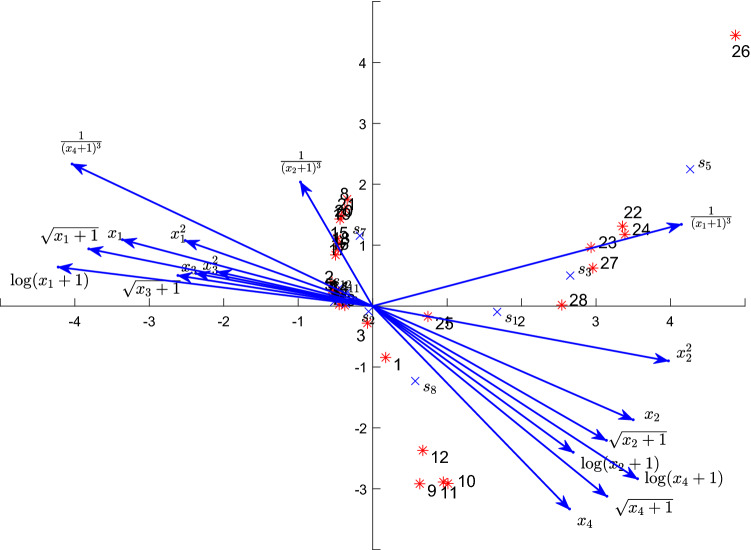
The biplot of the NCCA for spider species under the strategy 1. The “*” point represents a site, the number by the site represents the site number, the “$\times$” point represents a species, and $s_1$–$s_{12}$ represent the codes of 12 species, the arrows respectively represent the environmental variables and their nonlinear factors.

**Figure 2:**
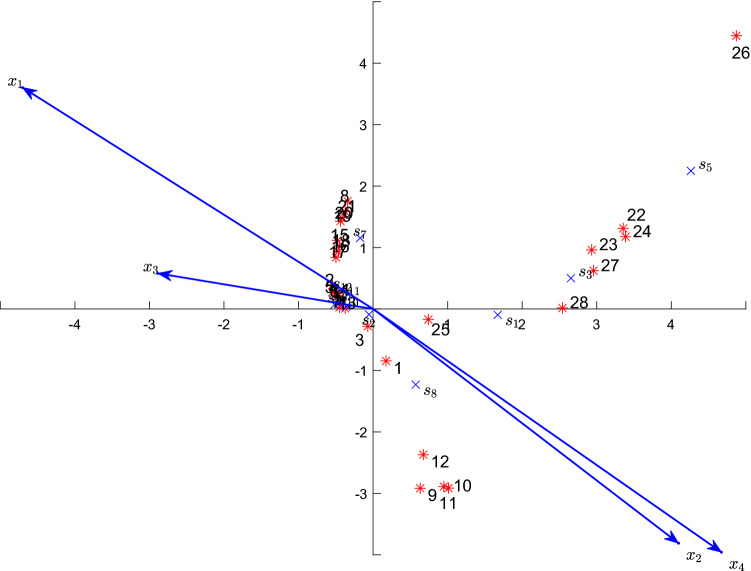
The biplot of the NCCA for the spider species under the strategy 2. The “*” point represents a site, the number by the site represents the site number, the “$\times$” point represents a species, and $s_1$–$s_{12}$ represent the codes of 12 species, the arrows respectively represent the environmental variables and their nonlinear factors.

By using the species scores, the site scores and the environmental factor scores in the columns “Axes 1” and “Axes 2” of Supplementary Table S1 , we plot the NCCA biplots as Figs. 1 and 2, according to the strategy 1 and the strategy 2, respectively. In both figures, the coordinates of the environmental factors are all magnified by 5 times. The dense area in the second quadrant of Fig. 1 or Fig.2 is drawn in in Supplementary Fig. S1

It should be noted that the position of an environmental factor in Fig. 2 is determined by the original variable and its nonlinear terms. Figure 2 is drawn according to the strategy 2, which is not the same as Fig. 1 drawn under the strategy 1. The strategy 2 mainly integrates the nonlinear terms of the environmental variables into the original environmental variables, which makes the positions of environmental factors drawn according to this strategy thus reflect the influence of the nonlinear terms of the original variable. By comparing the environmental variables $x_1$–$x_4$ in Figs. 1 and 2, we can find that the angle and the position of $x_3$ (the calamagrostis coverage) are still similar, while those of $x_1$ (the soil water content), $x_2$ (the reflection) and $x_4$ (the corynephorus coverage) are quite different. The results indicate the nonlinear terms of the environmental variable $x_3$ had less impacts on a species, but that of $x_1$, $x_2$ and $x_4$ had more impacts on a species. Under the action of the nonlinear terms of the environmental variables, the directions of $x_1$ and $x_4$ are almost the same, but the length of the arrow $x_4$ is still greater than that of the arrow $x_2$, which indicates $x_4$ is of more importance over $x_2$.

**Figure 3:**
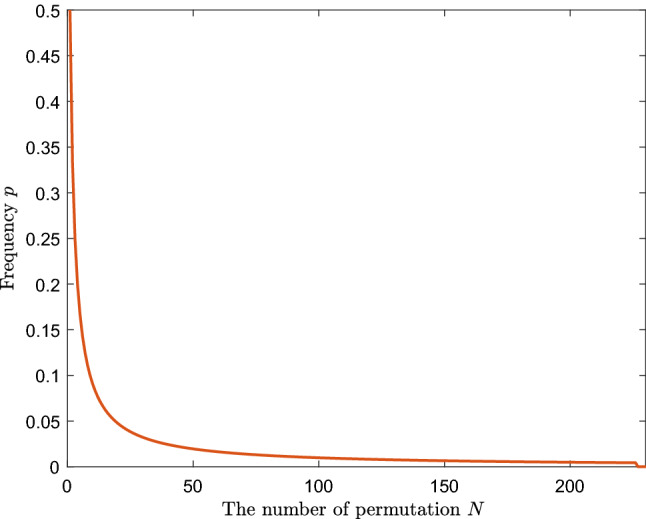
The relationship between frequency *p*-value and the number of permutations *N*.

As mentioned in Section of “Significance test in the NCCA”, the significance of the NCCA model can be evaluated by performing permutation tests. Taking the standard deviation $\delta =0.001$, $N=1000$ and $N_1=100$, after 227 permutations, the *p*-value reached 0.0044, then the test automatically stopped. Figure 3 shows the relationship between frequency *p*-value and the number of permutations *N*. The $p=0.0044<0.01$, which shows that the NCCA model is highly significant. Therefore, three models including the LCCA, the PCCA and the NCCA are all significant.

### Analysis and discussion

#### The distribution characteristics of the species and the sites on the biplot

The biplot of the NCCA can be used for analyzing the distribution characteristics of the species and the sites as a classical CCA. From Fig. 1 or Fig. 2, we can intuitively observed that the distributions of 12 species and 28 sites show two characteristics: one is grouped in the second quadrant, and the other is scattered in the first and fourth quadrants. The grouped sites include $2, 4 - 8$ and $13-21$, while the densely distributed species mainly include $s_4$, $s_6$, $s_7$ and $s_9-s_{12}$. Because each site point is located at the centroid of the species points near the site point, we can infer that the species may exist at a specific site on the biplot, and know that the abundance or the occurrence probability of a species decreases with the increasing of distance from the position of the species on a biplot. In Figs. 1 and 2, the species closest to the site 26 are $s_5$, $s_3$, $s_1$ and $s_8$ in proper order, which is consistent with the order of the number of these species on the site 26. The species $s_3$ is relatively close to the site 22, the site 23, the site 24, the site 27 and the site 28, which indicates that the species $s_3$ mainly appears at these site points. Indeed, the number of the species $s_3$ is the largest at these site points (see^22^, Table 1]). This distribution characteristic is basically consistent with the result calculated by the PCCA (see^22^, Fig. 5]). Therefore, just as the PCCA, the NCCA successfully restores the arch distorted by the LCCA, which represents the gradient in the correspondence analysis biplot. However, this recovery is realized by incorporating the nonlinear terms of the environmental variables in the species interpretation equation.

#### The influence of the environmental factors on the distribution of species

The species points and the environmental factor arrows in the biplot reflect the distribution of the species along each environmental factor. By projecting the species points onto an environmental factor arrow by using the vertical lines, the arrangement of these projection points on the environmental factor arrow represents the impact of the environmental factor on each species. For example, when the arrow refers to $x_1$ (the soil water content), we can roughly divide it into three parts: the positive direction of the arrow, near the origin and the negative direction of the arrow, which respectively represent the wetness, the moderate humidity and the dryness. Therefore, we can infer which species mainly appearing in the wet places, the dry places or the places with moderate humidity values. Accordingly, we know that all the sites points can be classified into three main groups. The first group representing the driest areas includes the site points $22 - 24$, and $26 - 28$ that are mainly highly positively correlated with the species $s_3$ and $s_5$. The second group representing the wet areas is the second quadrant of Fig. 1 or Fig. 2 and these areas mainly involve the site points $2, 4 - 8$, and $13 - 21$ that are of mainly high frequency correlation with the species $s_4$, $s_6$, $s_7$ and $s_9 - s_{12}$ (see Supplementary Fig. S1). The site points $1, 3, 9 - 12$ and 25 represent the medium humidity areas, which is mainly related to the value of the species $s_8$ of high frequency.

The more dispersed the arrangement of species along an environmental factor arrow, the more effective it is to distinguish the impact of environmental factors on the species distribution. In Fig. 1, the distribution of the projection points of some species along environmental factor arrows is obviously more dispersed than that of that along their original environmental factor,such as $x_1$ and $(x_1+1)^{-3}$, $x_4$ and $\log (x_4 + 1)$. Therefore, we can see in Fig. 2 that the arrow positions of the environmental variables $x_1$ and $x_4$ are shifted to the top-right when compare to those in Fig. 1, which can more effectively represent the impacts of $x_1$ and $x_4$ on the species distribution.

We know that the relative importance of the impacts of different environmental factors on species can be indicated by the arrow length, that is, the longer the arrow, the greater the impact. In Fig. 1, the arrow length of the nonlinear environmental factor $\log (x_1+1)$ ($\sqrt{x_1+1}$, ${(x_1+1)}^{-3}$) is longer than that of $x_1$, which is also true for $\log (x_4+1)$ ($\sqrt{x_4+1}$, ${(x_4+1)}^{-3}$) and $x_4$. Knowing from this, these nonlinear environmental factors have a greater impacts on the species distribution than their original environmental factors. The nonlinear factors of $x_3$ have the least impacts on the species distribution, the arrow length and positions of these nonlinear factors in Fig. 2 have thus hardly changed, while the arrow lengths of other environmental factors have been significantly lengthened.

#### The comparison of the NCCA with the LCCA and the PCCA

We know the three models, the LCCA, the PCCA and the NCCA are all highly significant over the spider species data according to the permutation test. If a model can explain more variances in $Y^*$, then its performance is better. The total variance in $Y^*$ is 1.92296. Although all 12 canonical axes generated by the NCCA are unable to explain the total variances in $Y^*$, they account together for $81.22888\%$ of total variances. As a comparing, this ratio for the PCCA is $80.3\%$, while that for the LCCA is only $43.80558\%$. The cumulative variances ratios of the first six canonical axes of the three models are listed in Table 7.

**Table 7:** Cumulative variance ratios of three CCA models for the spider species data.

Models	Canonical axes					
	Axis 1	Axis 2	Axis 3	Axis 4	Axis 5	Axis 6
LCCA (%)	28.35114	37.32036	42.41081	43.80558	43.80558	43.80558
PCCA (%)	32.70698	52.21410	65.87006	74.05001	76.63669	78.16481
NCCA (%)	36.89071	54.80844	68.83357	75.38650	77.83521	79.24605

The cumulative variance ratios of PCCA cited the data of the literature^22^.

Knowing from Table 7, the NCCA is the best one of the three models in the cumulative variance ratios of all canonical axes. For example, the first two canonical axes of the NCCA explain $54.80844\%$ of the total variance, which is significantly higher than the $37.32036\%$ obtained by the LCCA and the $52.21410\%$ obtained by the PCCA. Moreover, the cumulative variance ratio of the NCCA on the first six canonical axes reaches at $79.24605\%$.

The permutation test introduced in Section of “Significance test in the NCCA” can be employed to evaluate the variance difference significance of the three models. After 227 permutations, the significance *p* value of the variance difference between the NCCA and the LCCA is 0.0044, which indicates that the NCCA is more suitable than the LCCA for addressing the spider species data, and which is also true for the case between the NCCA and the PCCA.

## Conclusion

In ecology, the canonical correspondence analysis is widely used for analyzing the response variables (e.g the species variables) and the explanatory variables (e.g. the environmental variables), and the regression part of the classical CCA method is linear. However, the linear relationships are impractical in most cases. From the ecological perspective, the nonlinear describing of the relationship between the response variables and the explanatory variables is more accurate and practical than the LCCA model. From this, we propose a NCCA method based on fundamental CCA theories to solve the problem of more complex nonlinear relationships between the response variables and the explanatory variables.

In solving the problem of how to describe the nonlinear relationship between the response variables and the explanatory variables, the nonlinear transformations of the original explanatory variable data matrix is first performed, next, in order to avoid model over-fitting, we use the correlation and the LASSO regression to select the most appropriate explanatory variables and their nonlinear terms twice. Subsequently, the linear regression and the improved heuristic optimal quadratic approximation method are employed to fit the response variables data.

We solve the problem of how to describe the nonlinear factors with an ordination biplot. Under no many explanatory variables and their nonlinear terms, all of which can be shown on a biplot. Otherwise, only the original explanatory variables are drawn on a biplot, in which the coordinates are the complex correlation coefficients between the explanatory variables (including their related nonlinear terms) and the ordination axis. Because the coordinate of an explanatory variable arrow is determined by the complex correlation coefficient, this coordinate reflects the influence of the nonlinear factors.

We use the permutation method to test the significance of NCCA model. Numerical simulation shows that NCCA has good performance in preventing type I error and type II error. The application of our NCCA method to a real data-set (i.e. the predatory spider distribution data-set) also shows its effectiveness. Our method inherits the advantages of the PCCA, for example, it can recover the arch representing gradient distorted by the LCCA. Furthermore, the canonical axes in our method have greatly improved the ability to explain the variance of the original response variables, and the corresponding biplot can more effectively reflect the impacts of the explanatory variables on the distributions of the response variables.

Our method also solves the problem of nonlinear assumptions between the species and the explanatory variables by adding the nonlinear terms of some explanatory variables to the matrix of the original explanatory variables. However, accurately determining the nonlinear form in actual calculations is still difficult. In the practical examples in Section “Experiments”, we only calculated five special forms. With the increasing of nonlinear function forms, the computational complexity undoubtedly will greatly increase, which needs to be further studied in detail.

## Supplementary Information

Supplementary Information.

## Data Availability

The hunting spider dataset used and analyzed during this study are included in those published articles^22,37^.
