# Supplementary material for: Nonlinear canonical correspondence analysis and its application
Source: Sci Rep. 2023 May 9;13:7518. doi: 10.1038/s41598-023-34515-y (PMC10170120; doi:10.1038/s41598-023-34515-y)
Supplement: Supplementary file 1 — Supplementary Information. [file 41598_2023_34515_MOESM1_ESM.pdf]

Nonlinear canonical correspondence analysis and its application

Leru Zhou[1], Zhili Liu[2], Fei Liu[2], Jian Peng[2], and Tiejun Zhou[2,*]

[1]College of Resources and Environment, Xinjiang Agricultural University, Urumqi, 830052, China
[2]College of Information and Intelligence Science, Hunan Agricultural University, Changsha, 410128, China
[*]corresponding author.tj_zhou@hunau.edu.cn

ABSTRACT

This document is a supplement to the paper "Nonlinear canonical correspondence analysis and its application", including a table and a figure. Table S1 is the NCCA calculation result, and Figure S1 is the dense area in the second quadrant of Figure 1 and Figure 2 in the paper.

1 Table

Table S1. The NCCA results of the hunting spider data

	Canonical axes					
**Data type**	**Axes 1**	**Axes 2**	**Axes 3**	**Axes 4**	**Axes 5**	**Axes 6**
Eigenvalues (with respect to total variance of Y^* =1.922962487)						
	0.70939	0.34455	0.26970	0.12601	0.04709	0.02713
Variance ratios of Y^* (%)						
	36.89071	17.91774	14.02512	6.55294	2.44871	1.41084
Cumulative variance ratios of Y^* accounted for by Axes 1-6 (%)						
	36.89071	54.80844	68.83357	75.38650	77.83521	79.24605
Species scores						
s_1	1.67189	-0.09544	-0.02769	-0.47651	0.04104	-0.51331
s_2	-0.05476	-0.08900	0.02363	0.12114	0.23022	-0.00196
S_3	2.6533	0.50061	-0.054113	-1.11110	-0.077099	0.51444
S_4	-0.51704	0.069907	-0.58406	0.23596	-0.28871	0.087927
S_5	4.2637	2.2473	-1.0902	2.2431	0.15721	0.048818
S_6	-0.43198	0.13182	-0.33996	-0.0838	0.23524	0.067009
S_7	-0.17281	1.1554	2.1451	0.035021	0.44863	0.010389
S_8	0.57193	-1.2305	0.38264	0.32596	0.057512	0.076617
S_9	-0.45658	0.08844	-0.4509	-0.025416	0.29341	0.11851
S_{10}	-0.35913	0.038609	-0.38137	-0.02177	0.20715	-0.07335
S_{11}	-0.33717	0.23416	0.11024	0.10593	-0.18119	-0.043431
S_{12}	-0.44497	0.29982	0.022668	0.078443	-0.32119	0.16146
Site scores						
1	0.17418	-0.84493	0.03776	0.12711	-0.083566	-2.0091
2	-0.5459	0.38949	-0.53636	-0.14692	-0.23985	-0.07151
3	-0.071163	-0.28054	-0.20746	-0.068746	0.16828	-1.3642
4	-0.49577	0.2521	-0.6226	-0.12156	-0.02962	-0.28414
5	-0.54836	0.27055	-0.88792	-0.18513	1.4495	0.97785
6	-0.48284	0.19986	-0.35686	0.22906	-1.8891	1.2173
7	-0.45003	0.025948	-0.66018	0.039128	0.40871	0.32192
8	-0.34229	1.7559	3.919	0.10667	3.5105	0.14927

Continued on next page

Table S1 – *Continued from previous page*

Data type	Axes 1	Axes 2	Axes 3	Axes 4	Axes 5	Axes 6
9	0.63217	-2.9171	1.0347	1.6846	0.30464	1.6305
10	1.0112	-2.9145	1.1056	1.0223	-0.070583	0.81934
11	0.94907	-2.8894	1.0616	1.0997	0.088621	0.10941
12	0.67376	-2.37	0.82421	1.0565	0.30691	-0.50294
13	-0.37458	0.01038	-0.43566	0.068929	0.38398	0.24746
14	-0.46569	0.22127	-0.25384	0.14352	-1.3833	-0.17233
15	-0.4763	1.1156	1.6649	0.35228	-2.7093	-0.60689
16	-0.46564	0.93264	1.2654	0.33196	-2.1392	-0.25971
17	-0.49788	0.83911	0.89687	0.41299	-4.0368	-0.91697
18	-0.45357	1.0279	1.5686	0.38396	-2.3682	-1.2541
19	-0.43829	1.4294	2.597	0.25727	-0.43807	-0.23976
20	-0.41393	1.4799	2.8439	0.27826	-0.053289	-0.4964
21	-0.37425	1.5861	3.4117	0.25066	1.093	-0.29782
22	3.3555	1.3118	-0.60846	-3.381	-1.231	6.0507
23	2.9322	0.96192	-0.21803	-1.5162	-0.20609	-0.19975
24	3.3869	1.1758	-0.58769	-3.4771	-1.0313	4.8131
25	0.74155	-0.17081	0.2059	-1.5018	-1.5194	1.1742
26	4.8723	4.4462	-2.6848	8.8089	0.79198	3.1132
27	2.9568	0.62439	-0.48973	-3.3224	-0.47415	-7.3577
28	2.5402	0.016792	-0.022016	-4.19	-1.1405	-0.095995
Scores of environmental variables of strategy 1						
x_1	-0.6722	0.21805	-0.21126	0.15166	-0.10042	0.27246
x_2	0.69975	-0.37333	-0.12704	0.070818	0.20857	-0.29187
x_3	-0.47445	0.10704	-0.66474	-0.096883	0.51473	0.048121
x_4	0.52874	-0.66624	0.21772	0.08918	0.024574	-0.074123
$\sqrt{x_3+1}$	-0.52304	0.10051	-0.67761	-0.074267	0.48514	-0.009921
${x_2}^2$	0.79432	-0.17989	-0.082542	0.22097	0.11994	-0.25399
$\ln(x_1+1)$	-0.84462	0.12805	-0.14337	0.22916	-0.048272	0.23757
$\ln(x_2+1)$	0.53866	-0.48001	-0.32537	-0.063056	0.33887	-0.25272
$\sqrt{x_2+1}$	0.62778	-0.44096	-0.20599	0.0002057	0.27389	-0.28412
$\frac{1}{(x_4+1)^3}$	-0.80732	0.46657	-0.15006	-0.001524	-0.001353	-0.066587
$\sqrt{x_1+1}$	-0.76253	0.18784	-0.18524	0.18892	-0.079375	0.25695
$\ln(x_4+1)$	0.71134	-0.56705	0.1856	0.030477	0.010784	0.0062992
${x_3}^2$	-0.417	0.11269	-0.63704	-0.11492	0.51225	0.11717
$\sqrt{x_4+1}$	0.62911	-0.62445	0.20489	0.053968	0.016866	-0.032258
$\frac{1}{(x_1+1)^3}$	0.82924	0.26828	-0.097858	-0.30663	-0.081898	-0.26101
${x_1}^2$	-0.50344	0.21524	-0.22669	0.093359	-0.11117	0.28435
$\frac{1}{(x_2+1)^3}$	-0.19393	0.40729	0.69901	0.1302	-0.19481	0.056366
${x_1}^2$	-0.51534	0.18751	-0.18967	0.073281	0.27996	-0.14511
e^{-x_2}	-0.21556	0.38704	0.80012	0.069297	-0.010959	-0.15873
$\frac{1}{(x_2+1)^3}$	-0.21419	0.38662	0.80166	0.061327	0.012252	-0.11354
Scores of environmental variables of strategy 2						
x_1	-0.94070	0.72053	-0.34071	0.32287	-0.66269	0.78745
x_2	0.81951	-0.76222	-0.80720	0.58853	0.58478	-0.34785
x_3	-0.57753	0.11584	-0.68634	-0.15291	0.55224	0.35646
x_4	0.93458	-0.79143	0.29007	0.64412	0.11540	-0.33065

Note: The scores are calculated based on the scale type 2.

2 Figure

Figure S1 is the dense area in the second quadrant of Figure 1 and Figure 2 in the paper.

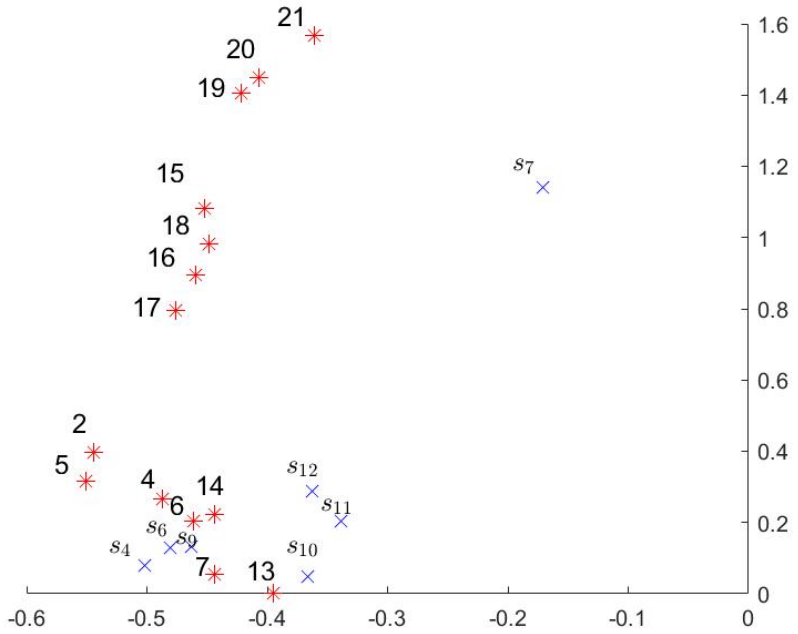

Figure S1. The sub-graph in the second quadrant of the biplot Figure 1 of the NCCA for the spider species under the strategy 2. The "*" point represents a site, the number by the site represents the site number, the "$\times$" point represents a species, and $s_1 - s_{12}$ represent the codes of 12 species.
